# Editorial: Molecular mechanisms and therapeutic strategies of head and neck disease

**DOI:** 10.3389/fcell.2026.1859260

**Published:** 2026-05-12

**Authors:** Yu-Jing Song, Xu Zhang, Jian-Hua Wang, Wei-Wei Deng, Lei-Lei Yang

**Affiliations:** 1 Department of Stomatology, The First Affiliated Hospital of Zhengzhou University, Zhengzhou, China; 2 State Key Laboratory of Oral and Maxillofacial Reconstruction and Regeneration, Key Laboratory of Oral Biomedicine Ministry of Education, Hubei Key Laboratory of Stomatology, School and Hospital of Stomatology, Frontier Science Center for Immunology and Metabolism, Wuhan University, Wuhan, China; 3 Taikang Center for Life and Medical Sciences, Wuhan University, Wuhan, China

**Keywords:** biomarker-driven strategies, head and neck squamous cell carcinoma (HNSCC), immune checkpoint inhibitors (ICIs), immune evasion, therapeutic, tumor microenvironment

Head and neck squamous cell carcinoma (HNSCC) remains a clinical challenge, characterized by profound anatomical complexity and therapeutic recalcitration. While standard curative-intent modalities for locoregionally advanced disease rely on surgery and multimodality therapy, the integration of immune checkpoint inhibitors (ICIs) is fundamentally reshaping the therapeutic landscape (Dunn et al., 2026). Pivotal phase III studies have demonstrated that perioperative and adjuvant PD-1 blockade with pembrolizumab or nivolumab significantly reduces the risk of relapse in patients with high-risk resectable head and neck squamous cell carcinoma (Bourhis et al., 2026; Uppaluri et al., 2025).

Nevertheless, emerging insights into the HNSCC tumor microenvironment (TME) underscore that durable responses to PD-1 blockade remain confined to a limited subset of patients (Ruffin et al., 2023). Such biological heterogeneity challenges the “one-size-fits-all” application of ICI therapy. The profound toxicity and financial burden of prolonged ICI regimens underscore an urgent need for precise, biomarker-driven strategies. This Research Topic brings together eight studies that examine the molecular features, immune microenvironment, and predictive biomarkers relevant to refining treatment selection in HNSCC.

The mechanistic basis of therapeutic resistance in HNSCC is multifactorial, encompassing both structural alterations in the tumor barrier and dynamic reprogramming of the immune infiltrate. Overcoming inherent therapeutic resistance demands a profound understanding of structural barrier components and chemokine networks shaping the TME. Dang et al. identified the tight junction protein CLDN10 as a critical prognostic biomarker, revealing its severe downregulation in human papillomavirus (HPV)-negative HNSCC. Crucially, their work shifts the immunological focus toward humoral immunity, demonstrating that preserved CLDN10 expression dictates robust infiltration of CD20^+^ and CD38^+^ B-cells, conferring a significant survival advantage. Conversely, tumor progression is actively fueled by chemokine-driven epithelial-mesenchymal transition (EMT). Liu et al. elucidated how pathological CCL26 secretion from cancer-associated fibroblasts establishes an immunologically “cold tumor” microenvironment. Elevated CCL26 downregulates epithelial markers while upregulating mesenchymal markers like Vimentin, systematically repelling cytotoxic CD8^+^ T-cells and downregulating immune checkpoints, rendering tumors inherently resistant to standard blockade. Together, these findings identify structural barrier loss and chemokine-induced EMT as convergent drivers of immune evasion in HNSCC.

Aggressive HNSCC proliferation is also sustained by dysregulation of conserved intracellular cascades. Yang and Li. provided an analysis of the atypical Hippo signaling pathway, detailing how core kinases (TAOK, MAP4K, NDR1/2) operate independently of the classical MST1/2 axis to drive YAP/TAZ nuclear translocation. By mapping the extensive crosstalk between the atypical Hippo network and Wnt/β-catenin pathways, they identify direct YAP/TAZ-TEAD complex inhibition as a potential strategy for overcoming multi-drug resistance. At single-cell resolution, Zhao et al. mapped the spatial crosstalk between Natural Killer (NK) cells and Tumor-Associated Macrophages (TAMs). Demonstrating mutual exclusivity between effector IL32+ NK cells and immunosuppressive APOE + TAMs, they developed the 23-gene CINT prognostic signature, providing a mechanistically grounded tool to accurately predict PD-1/PD-L1 blockade efficacy. Collectively, these findings underscore that therapeutic response in HNSCC is shaped jointly by intracellular signaling networks and the spatial organization of immune effector cells.

Translating these mechanistic insights into clinical practice requires robust validation in real-world treatment cohorts across distinct disease stages. Ouyang et al. validated the highly intensive TPCE regimen (tislelizumab, nab-paclitaxel, carboplatin, and cetuximab), reporting an objective response rate of 82.1%. Importantly, their real-world evidence established baseline inflammatory markers, specifically the hemoglobin-albumin-lymphocyte-platelet (HALP) score and dynamic neutrophil-to-lymphocyte ratio (NLR) reductions, as prognostic indicators of immunotherapy response. In parallel, Li et al. assessed the safety and efficacy of substituting solvent-based taxanes with nab-paclitaxel to avoid immunosuppressive corticosteroids, achieving a response rate of 60.0% with the toripalimab, nab-paclitaxel, and platinum combination. For definitive concurrent chemoradiotherapy (CCRT), Zhang et al. examined the predictive value of biomarkers in locally advanced HNSCC, confirming that high PD-L1 expression (CPS ≥ 20) is associated with improved CCRT outcomes. Furthermore, their analysis shows that deficient mismatch repair (dMMR) tumors, while initially less responsive to radiotherapy, remain susceptible to subsequent immunotherapy due to sustained immunogenicity. Overall, these studies demonstrate that treatment intensification and biomarker-guided patient selection are key to improving outcomes in both frontline and salvage settings for advanced HNSCC.

Despite these therapeutic advances, the pattern of post-treatment relapse also carries implications for trial design and surveillance. Qu et al. analyzed a cohort of over 7,500 R/M HNSCC patients, revealing that high-risk treatments like primary chemoradiation or trimodality therapy paradoxically correlate with highly aggressive, rapid relapse (median 6–7 months) and dismal survival. This finding exposes a critical flaw in modern clinical trial designs, which frequently exclude these highly vulnerable, early-relapsing patients, potentially skewing efficacy data.

In summary, the management of HNSCC is moving toward more individualized approaches that consider both tumor biology and the immune contexture. The studies collected here illustrate how insights into the Hippo pathway, chemokine-mediated immune exclusion, and spatial TME organization can inform prognostic models such as the CINT signature. Ultimately, improving outcomes in HNSCC will depend on integrating these adaptive methodologies and dynamic biomarkers to personalize both the escalation and de-escalation of therapeutic regimens, thereby maximizing survival while mitigating treatment-related morbidity.
